# Congenital Club-Foot

**Published:** 1887-06

**Authors:** 


*Congenital Club-foot.* By ROBERT WILLIAM PARKER. Pp. 104. London: H. K. LEWIS. 1887.

By honest work Mr. Parker has established the right to be heard as an authority on club-foot. This book presents in short and readable form his matured conclusions as to the pathology and the treatment of this deformity. He endeavours to establish the following propositions:

"That certain of the tarsal ligaments are relatively short, owing to developmental malposition of the bones between which they lie." To malposition we should add "and malformation": the bones are under-developed on one side, thus the ligaments on this side are shortened.

"That this anatomical condition is CONSTANT, and is the chief impediment to the reduction of the deformity." With this we heartily agree.

It is argued therefrom—

"That simple tenotomy leaves the major part of the anatomical condition untouched."

"That the relapses which so frequently occur after tenotomy, and even after the prolonged use of mechanical appliances, are largely due to the unyielding nature of these ligaments; and

"That the remedy is to be sought in DIVISION OF THESE STRUCTURES."

With the third and fourth propositions we also heartily agree. To the fourth we should add, "along with manipulation and encasement of the foot in unyielding bandages."

Probably the final blow has been dealt to tenotomy for congenital club-foot. Following sound pathology, we know that over-pressure prevents growth of bone, and under-pressure permits growth: if a club-foot is forcibly manipulated into the straight position, the bones on the convexity are prevented from growing, while the compressed bones on the concavity are permitted to grow. If the bones grow the ligaments will grow, and the process may be managed inside a plaster casing. If the child is too old, then division of ligaments will permit the out-folding of the foot. To divide the calcaneo-scaphoid ligament is to greatly weaken the arch of the foot, and may ultimately predispose to one of the most troublesome forms of flat-foot. Therefore, only on failure of manipulation and immobility should we resort to this expedient. Division of the plantar fascia is very frequently of great value as supplementary to forcible manipulation; but we should hesitate before dividing the ligament which practically supports the keystone of the arch of the foot.

We can heartily commend Mr. Parker's book. His pathology is sound, his reasoning is acute and convincing; and if his treatment has swung a little too far on the side of the heroic, and has partly overlooked the character of results got by simpler methods, it has the merit of striking at the root of the real cause of the disease.

